# Expert-Level Intracranial Electroencephalogram Ictal Pattern Detection by a Deep Learning Neural Network

**DOI:** 10.3389/fneur.2021.603868

**Published:** 2021-05-03

**Authors:** Alexander C. Constantino, Nathaniel D. Sisterson, Naoir Zaher, Alexandra Urban, R. Mark Richardson, Vasileios Kokkinos

**Affiliations:** ^1^Brain Modulation Lab, Department of Neurological Surgery, University of Pittsburgh School of Medicine, Pittsburgh, PA, United States; ^2^Department of Neurosurgery, Massachusetts General Hospital, Boston, MA, United States; ^3^Department of Neurology, University of Pittsburgh School of Medicine, Pittsburgh, PA, United States; ^4^University of Pittsburgh Comprehensive Epilepsy Center, Pittsburgh, PA, United States; ^5^Harvard Medical School, Boston, MA, United States

**Keywords:** epilepsy, responsive neurostimulation, seizure detection, ictal pattern, deep learning

## Abstract

**Background:** Decision-making in epilepsy surgery is strongly connected to the interpretation of the intracranial EEG (iEEG). Although deep learning approaches have demonstrated efficiency in processing extracranial EEG, few studies have addressed iEEG seizure detection, in part due to the small number of seizures per patient typically available from intracranial investigations. This study aims to evaluate the efficiency of deep learning methodology in detecting iEEG seizures using a large dataset of ictal patterns collected from epilepsy patients implanted with a responsive neurostimulation system (RNS).

**Methods:** Five thousand two hundred and twenty-six ictal events were collected from 22 patients implanted with RNS. A convolutional neural network (CNN) architecture was created to provide personalized seizure annotations for each patient. Accuracy of seizure identification was tested in two scenarios: patients with seizures occurring following a period of chronic recording (scenario 1) and patients with seizures occurring immediately following implantation (scenario 2). The accuracy of the CNN in identifying RNS-recorded iEEG ictal patterns was evaluated against human neurophysiology expertise. Statistical performance was assessed *via* the area-under-precision-recall curve (AUPRC).

**Results:** In scenario 1, the CNN achieved a maximum mean binary classification AUPRC of 0.84 ± 0.19 (95%CI, 0.72–0.93) and mean regression accuracy of 6.3 ± 1.0 s (95%CI, 4.3–8.5 s) at 30 seed samples. In scenario 2, maximum mean AUPRC was 0.80 ± 0.19 (95%CI, 0.68–0.91) and mean regression accuracy was 6.3 ± 0.9 s (95%CI, 4.8–8.3 s) at 20 seed samples. We obtained near-maximum accuracies at seed size of 10 in both scenarios. CNN classification failures can be explained by ictal electro-decrements, brief seizures, single-channel ictal patterns, highly concentrated interictal activity, changes in the sleep-wake cycle, and progressive modulation of electrographic ictal features.

**Conclusions:** We developed a deep learning neural network that performs personalized detection of RNS-derived ictal patterns with expert-level accuracy. These results suggest the potential for automated techniques to significantly improve the management of closed-loop brain stimulation, including during the initial period of recording when the device is otherwise naïve to a given patient's seizures.

## Introduction

Since its clinical establishment in the early twentieth century, intracranial electroencephalography (iEEG) has become the fundamental modality for evaluation and subsequent management in epilepsy surgery (1–4). Recorded either with the use of subdural electrodes (5) or stereotactic electroencephalography (sEEG) (6), the iEEG allows for localization of the epileptogenic zone or the epileptogenic network giving rise to seizures (7, 8). Computer-assisted signal processing methodologies became popular in the field to support the tedious task of seizure onset localization (9–11).

Deep learning methodologies have been successful in the medical field due to their efficiency in information extraction from raw data (12). One of the most recently established approaches to machine-learning is the convolutional neural network (CNN) model. CNNs are artificial neural networks with multiple consecutive layers that perform convolutions in a hierarchical fashion (13, 14). They are considered to be the deep learning model of choice in applications that require processing of multiple array data, as they can successfully identify local conjunctions in data and build high-level features from low-level ones (15). In the brain-related sciences and clinical fields, neural networks have become a core entity of brain-computer interfaces (16–23), assisted diagnosis and rehabilitation for brain disorders (24–27), and allowed methodological improvements in neuroscience (28–31). For electroencephalographic (EEG) data analysis specifically, deep learning by means of CNNs has been applied for feature extraction purposes (32–34), prediction of cognitive performance (35, 36), and identification of evoked potentials (37).

In recent years, deep learning has been applied in extracranial EEG data to facilitate seizure detection in adult (38–41), children (42), and neonatal populations (43), as well as to identify interictal EEG features (44, 45). Fewer studies, have used deep learning to detect seizures from iEEG data (46). Machine learning approaches have also been used to link extracranial EEG with ECoG discharges (47), predict epileptic seizures (41, 48), and design seizure-detection embedded systems (49). The studies aiming at developing deep learning approaches using intracranial seizure data derived from pre-surgical evaluations for epilepsy are highly limited by the small number of recorded seizures available per patient.

More recently, neuromodulation by the Food and Drug Administration (FDA)-approved RNS System has been used in the U.S.A. as an alternative minimally invasive and personalized therapy for patients with pharmacoresistant focal epilepsy (50). The RNS system is an implantable closed-loop electrical stimulation device that applies electrical stimulation to epileptogenic tissue upon detection of ictal patterns (51–54). The electric current applied locally over the seizure onset areas affects the progress of the detected ongoing ictal patterns by acutely causing their attenuation (55) or by chronically inducing changes in the epileptic synchronization and neuronal recruitment properties of the underlying epileptogenic tissue (56). For the first time in the history of iEEG, RNS allows the recording of iEEG epochs over long periods of time, resulting in the accumulation of hundreds and often thousands of iEEG epochs per patient per year. However, a study to evaluate the efficiency of CNNs in large intracranial RNS-derived seizure datasets remains lacking. As a consequence, the development of reliable automated seizure detection methods is urgently needed to support routine clinical evaluation of RNS patients, as well as to facilitate analytics for personalized treatment (57). Our study addresses this need and evaluates the efficiency of deep learning methodology in detecting iEEG ictal patterns using a large RNS-derived dataset of ictal patterns.

## Method

### Patients

Patients included in this study suffered from focal epilepsy, diagnosed according to current ILAE criteria (58, 59). Patients underwent investigative intracranial recording procedures, either by subdural electrodes, or by robotic-assisted stereotactic EEG, to identify the focus and extent of their epileptogenic zone. After a review of all available patient data during weekly multidisciplinary epilepsy conferences and consideration of available therapeutic options, closed-loop neurostimulation therapy (RNS, NeuroPace, Mountain View, CA, USA) was recommended. Our patients were implanted with the RNS system between January 2015 and June 2018, and the use of their data for this study was approved by the University of Pittsburgh Institutional Review Board (IRB).

### RNS Implantation

RNS leads were implanted as closely as possible to the recorded and/or hypothesis-derived epileptogenic regions (Supplementary Figure 1). Patients with a diagnosis of neocortical epilepsy onset were implanted either with strips placed over the focus, or depth leads placed through the focus, or a combination of both. Patients with a diagnosis of malformations of cortical development were implanted with depth leads across the posterior-anterior direction of the lesion. Patients with a diagnosis of mesio-temporal epilepsy were implanted with depth electrodes placed across the posterior-anterior axis of the hippocampus. Patients with a diagnosis of idiopathic generalized epilepsy were implanted in the thalamus by oblique depth electrodes targeting the centro-median nucleus. Assessment of electrode locations was performed by fusion of the post-surgical CT with the pre-surgical MRI.

### Data Acquisition

iEEG data recorded from the RNS system were obtained from NeuroPace. Additional RNS-related metadata, including recording, detection and stimulation settings, were collected directly from the NeuroPace Patient Data Management System (PDMS) using purpose-built software. Recordings consisted exclusively of 90 s duration, 4-channel ECoGs, online band-pass filtered at 4–125 Hz, sampled at 250 Hz and digitized by a 10-bit ADC. iEEG channel derivations were bipolar between neighboring electrode contacts (Supplementary Figure 1), grounded to the case of the RNS pulse generator. All electrode impedances measured below 1 kOhm for all recordings. Both scheduled and detection-triggered iEEG recordings were obtained and used in this study. Scheduled recordings were triggered by the RNS device's onboard clock to occur either every 12 or 24 h and offered a continuous sampling of spontaneous neurophysiologic activity. Detection triggered recordings were initiated by one of the onboard closed-loop algorithms. Patients were instructed to download their raw iEEG data daily to a local computer, through a transcutaneous telemetry wand, which was in turn uploading the recordings to the NeuroPace PDMS on a weekly basis. Immediately post-implantation, the device was set to passive recording mode for ~1 month, during which no stimulation was delivered in order to record baseline activity (baseline epoch). Once baseline activity was reviewed, stimulation parameters were configured and activated. During the rest of the post-implantation period the device delivered detection-triggered stimulation therapy and parameters were periodically modified in subsequent clinic visits based on evaluation of seizure control status. The time interval during which RNS parameters remain unchanged is referred to as programming epoch.

### Data Labeling

In accordance with established clinical practice, iEEG ictal patterns were visually identified by an experienced epilepsy surgery neurophysiologist (V.K.) and in turn confirmed by a board-certified epileptologist (N.Z.). The evaluation process was not influenced by and did not take into account the “long-episode” detections of the RNS system. The onset of ictal patterns was annotated by a cursor marker. The term “ictal pattern” is used instead of the term “seizure,” as the device provides no information regarding the clinical manifestation of the electrographic events. The iEEG ictal pattern onset was defined as the point in time after which the iEEG recording background was no longer interictal and was followed by a paroxysmal discharge of ictal features with evolution in frequency and morphology over time. Interictal background was evaluated from scheduled recordings that did not contain iEEG ictal patterns.

### Data Augmentation

To reduce overfitting of the model to the training data, we applied label-preserving transformations to iEEGs in the training set (60). We padded the iEEGs with 30 s of zero-voltage measurements before and after the recording, and then chose a 90-s crop uniformly at random. We also rescaled the data by multiplying each signal by a factor between 0.8 and 1.2, chosen uniformly at random for each iEEG. The network was evaluated on untransformed iEEGs from separate validation sets.

### Model Architecture and Training

**Figure 1:**
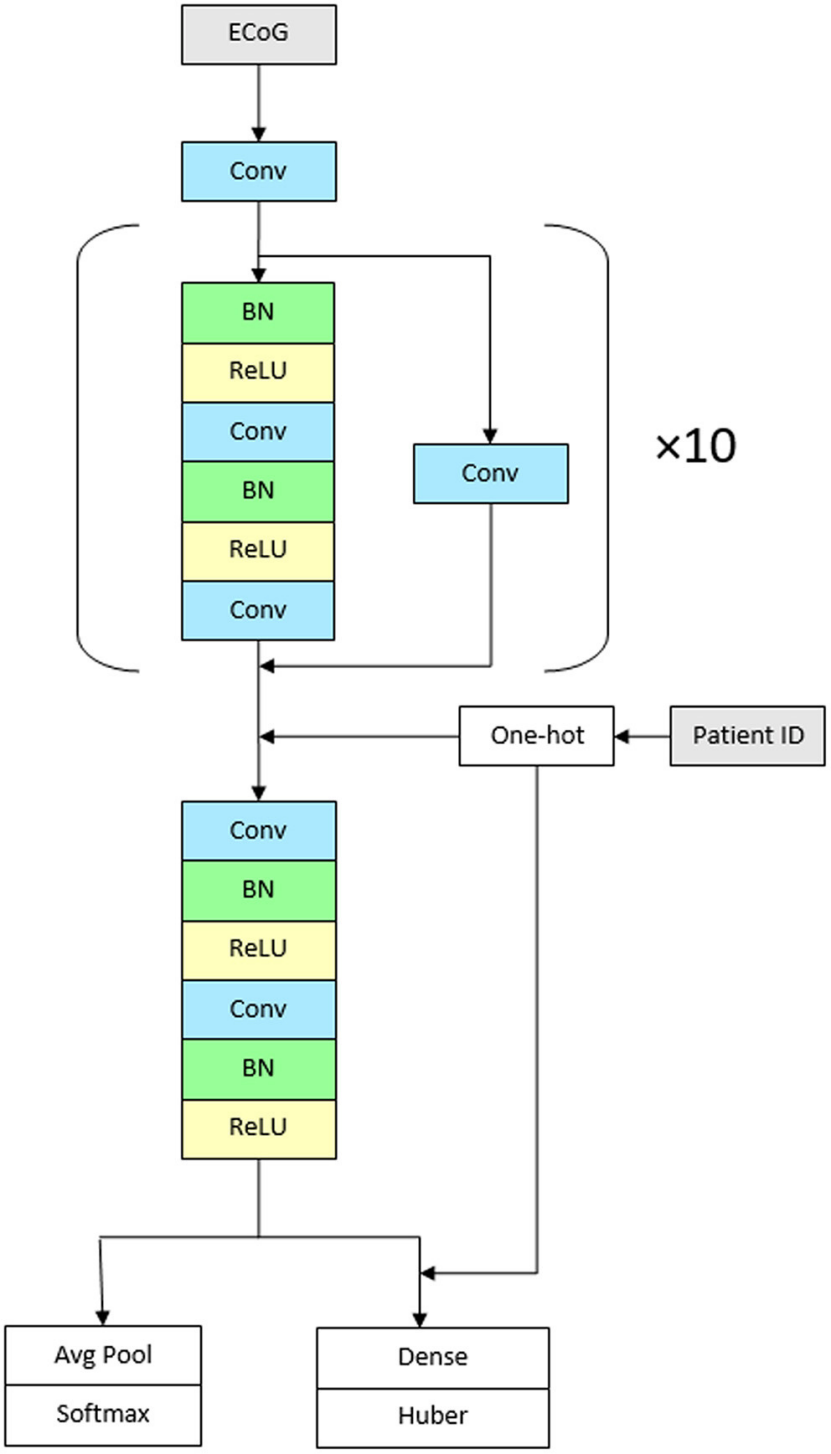
Architecture of the network. The CNN contains 23 convolutional layers with residual connections. The network takes as input a time-series of intracranial voltage measurements and a patient identifier. Before each convolutional layer, batch normalization is applied followed by rectified linear activations. The network contains 11 residual blocks with 2 one-dimensional convolutional layers per block. The convolutional kernel size is 16. The number of filters is 16 in the first layer and increases linearly to 116 in the penultimate residual block. The network outputs two predictions: a probability that the recording contains an ictal pattern, and the onset time of the ictal pattern in seconds (Conv, convolutional layer; BN, batch normalization; ReLU, rectified linear activations).

We used a convolutional neural network with high-level architecture shown in Figure 1. The network contains 23 convolutional layers with residual connections to make optimization of such a deep network tractable (61). The network takes as input a time-series of intracranial voltage measurements and a patient identifier. The patient identifier facilitates personalized ictal pattern prediction by allowing the network to make predictions conditioned to a particular patient. The network outputs two predictions: a probability that the recording contains an ictal pattern, and the onset time of the ictal pattern in seconds. We jointly optimize both losses using a hybrid loss function. Defining *s* ∈ {0, 1} as ictal pattern label, ŝ as predicted ictal pattern probability, t as ictal pattern onset time, and $\hat{t}$ as predicted ictal pattern onset time, the loss for one example is:

$$\begin{array}{l} L\left(s,\hat{s},t,\hat{t}\right)=crossentropy\left(s,\hat{s}\right)+0.1\;huber\left(t,\hat{t}\right) \end{array}$$

where

$$\begin{array}{l} crossentropy\left(s,\hat{s}\right)=-s\log\left(\hat{s}\right)\;-\left(1-s\right)\log\left(1-\hat{s}\right) \end{array}$$

and

$$\begin{array}{l} \text{huber}\left(t,\hat{t}\right)=\{\begin{array}{cc} \frac{1}{2}{\left(t-\;\hat{t}\right)}^{2} & \text{for }|t-\;\hat{t}|\leq 1\\ |t-\hat{t}|-\frac{1}{2} & \text{otherwise} \end{array} \end{array}$$

The network contains 11 residual blocks with 2 one-dimensional convolutional layers per block. The convolutional kernel size is 16. The number of filters is 16 in the first layer and increases linearly to 116 in the penultimate residual block. At that point, the filters are concatenated with one-hot encoded patient IDs followed by the final residual block. At the first convolutional layer and at the start of every other residual block, the stride is 2, which down samples the data by a factor of 2 every other residual block. Alternating residual connections also use a stride of 2 in their convolution.

Before each convolutional layer, we apply batch normalization (62) followed by rectified linear activations (63). We initialize the weights (64) and train the network using stochastic gradient descent for 20 epochs with a batch size of 128. We use cyclical learning rates (65) by cycling the learning rate from 0.1 to 0.025 every 4 epochs. For the final two epochs, the learning rate is held at 0.025. Experiments were conducted on Nvidia Tesla K80 accelerators using TensorFlow 1.13.

Accuracy is evaluated by concordance with expert identification, as well as the empirical time constraint for detecting the ictal pattern onset at an interval of less than ±5 s from the expert onset marking, corresponding to half the 20 s EEG review page typically used in clinical routine.

Annotated iEEG ictal patterns from the dataset were partitioned into training and testing sets; the training set was used to introduce data and the testing set to measure algorithm performance. We decided to create the following two experimental scenarios that correspond to actual clinical situations: (1) when a patient already implanted with RNS moves his epilepsy care to a new center, and the new center receives all prior RNS recordings for analysis, or when an RNS clinic physician moves to a new center, where a list of RNS patients is already registered for care (scenario 1) and, (2) the situation when a new patient is implanted (scenario 2). To test the 1st scenario on previously unseen data of patients that have already been recorded for some time (scenario 1), cross-validation was performed using leave-one-out methodology. This was done by training and evaluating the network on all ictal patterns obtained from all except one patient. Ictal patterns from this “hold-one-out” patient were randomly selected to form a seed set that was then used to train the CNN. The size of seed set was increased from *n* = 0 to *n* = 30 at increments of 5. In addition, each seed set was paired with an equal number of interictal epochs free from ictal patterns; for this purpose, non-ictal scheduled recordings from the “hold-one-out” patient were used to pair seed set recordings containing ictal patterns from the same patient. We then evaluated CNN accuracy on the held-out data, i.e., ictal patterns of the “hold-one-out” patient not used in the seed set. This experiment was repeated in separate iterations for all patients. To test the 2nd scenario on data of newly implanted patients (scenario 2), we used the “hold-one-out” patient's earliest available consecutive ictal patterns as seed set, corresponding to the baseline and early stimulation programming epochs, and then trained and evaluated the network as in scenario 1. Testing in both scenarios was performed in 12/22 patients for which at least *n* + 5 iEEG ictal patterns (i.e., # of ictal patterns > 35) were available (Table 1). The data of the rest of the patients were not used for testing. We trained our CNN to classify each iEEG epoch as containing an ictal pattern vs. no ictal pattern. Binary classification or detection accuracy was evaluated using area under precision-recall curve (AUPRC), which incorporates positive-predictive value to adjust for the significant class imbalance in our data set. For regression accuracy (predicting the time at which an ictal pattern begins), we used mean absolute error.

**Table 1:** Patient demographics and RNS data features.

**Patient**	**Age**	**Gender**	**Implantation site**	**# of days with RNS**	**# of iEEG files**	**# of ictal patterns**
1	21	F	Thalamus	95	349	11
2	22	M	Developmental malformation	166	333	13
3^*^	42	F	Neocortex	677	1,682	430
4^*^	22	F	Hippocampus	393	1,316	452
5^*^	39	F	Hippocampus	314	716	73
6	29	M	Developmental malformation	152	294	9
7^*^	22	F	Hippocampus	461	1,396	567
8^*^	34	F	Neocortex	600	1,172	113
9^*^	24	M	Neocortex	425	1,304	258
10	19	F	Thalamus	355	16	5
11^*^	39	F	Developmental malformation	297	834	720
12^*^	31	M	Hippocampus	261	443	47
13	46	M	Hippocampus	17	46	4
14	53	M	Neocortex	42	90	1
15	22	M	Thalamus	171	529	13
16^*^	63	F	Neocortex	732	4,110	2,057
17	35	F	Neocortex	19	95	4
18	37	M	Neocortex	735	508	20
19	31	F	Thalamus	73	299	9
20^*^	38	F	Hippocampus	202	522	93
21^*^	30	M	Hippocampus	376	796	159
22^*^	47	F	Developmental malformation	783	1,518	168
			Total	7,346	18,368	5,226

* *Patients with > 35 iEEG ictal patterns used in the testing dataset*.

### Statistical Analyses

Kruskal-Wallis-tests were used to compare AUPRC results between implant location groups, with an a priori level of significance set to 0.05. All analyses were performed using R 3.1.6 (R Foundation for Statistical Computing, Vienna, Austria) and all data was stored on Microsoft SQL Server 2012 R2 (Microsoft Corporation, Redmond, Washington, USA).

## Results

In this study we used a large RNS-derived intracranial dataset comprised of 5,226 ictal pattern events, marked and verified by consensus by two epilepsy experts (agreement on 99.8% of markings), in 18,368 epochs of ~90 s duration from 22 epilepsy patients implanted with RNS, corresponding to a total of 7,346 days of intracranial recording. The mean total post-implantation recording period was 47.7 ± 7.5 weeks (minimum 2.4 weeks, maximum 111.9 weeks). The mean patient age was 33.9 ± 2.5 years and 13 (59.1%) were women (Table 1).

**Figure 2:**
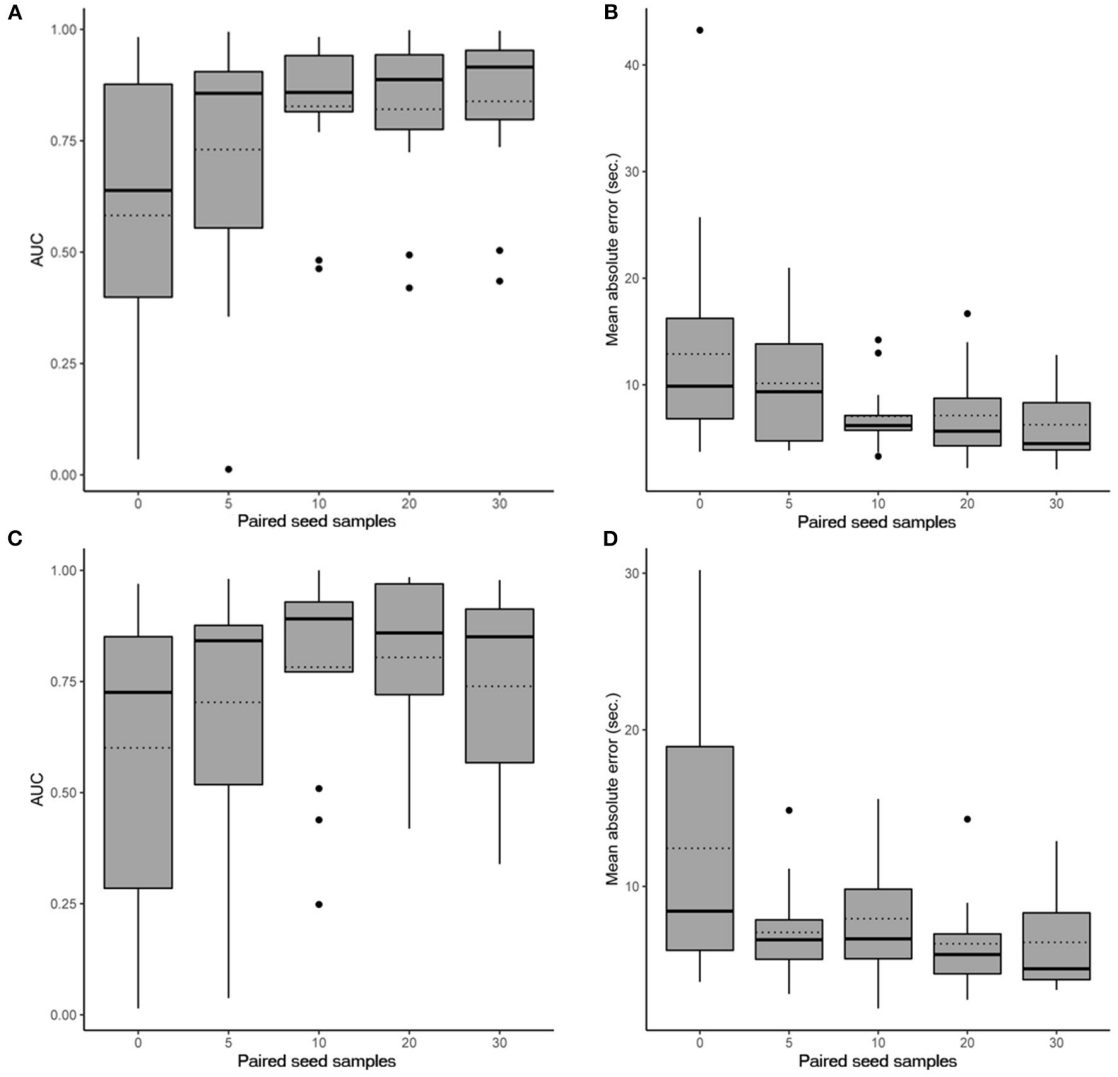
Performance evaluation of the CNN. Box plots show the 25th and 75th percentiles, median (solid), mean (dotted), minimum, and maximum values with outliers shown as dots. **(A)** AUPRC results (accounting for all past patients) for different numbers of paired seed data for our CNN in scenario 1 when detecting iEEG ictal patterns vs. non-ictal patterns. **(B)** Absolute mean regression error results for our CNN in scenario 1. **(C)** AUPRC results (accounting for all past patients) for different numbers of paired seed data for our CNN in scenario 2 when detecting iEEG ictal patterns vs. non-ictal patterns. **(D)** Absolute mean regression error results for our CNN in scenario 2.

In scenario 1, the CNN achieved a maximum mean binary classification AUPRC of 0.84 ± 0.19 (95%CI, 0.72–0.93) (Figure 2A) and mean regression accuracy of 6.3 ± 1.0 s (95%CI, 4.3–8.5 s) at 30 seed samples (Figure 2B). In scenario 2, maximum mean AUPRC was 0.80 ± 0.19 (95%CI, 0.68–0.91) (Figure 2C) and mean regression accuracy of 6.3 ± 0.9 s (95%CI, 4.8–8.3 s) at 20 seed samples (Figure 2D). However, we obtained near-maximum accuracies at seed size of 10 in both scenarios (Figure 2), suggesting significant transference between patients at a small seed size.

Sub-analysis by brain region implanted in scenario 1 showed an AUPRC of 0.88 ± 0.08 (95%CI, 0.83–0.93) for the hippocampus, 0.92 ± 0.09 (95%CI, 0.88–0.96) for developmental anomalies, and 0.73 ± 0.24 (95%CI, 0.60–0.86) for the neocortex (*p* = 0.35). In scenario 2, the AUPRC was 0.89 ± 0.09 (95%CI, 0.85–0.94) for the hippocampus, 0.93 ± <0.01 (95%CI, 0.93–0.93) for developmental anomalies, and 0.59 ± 0.29 (95%CI, 0.44–0.75) for the neocortex (*p* = 0.15).

**Figure 3:**
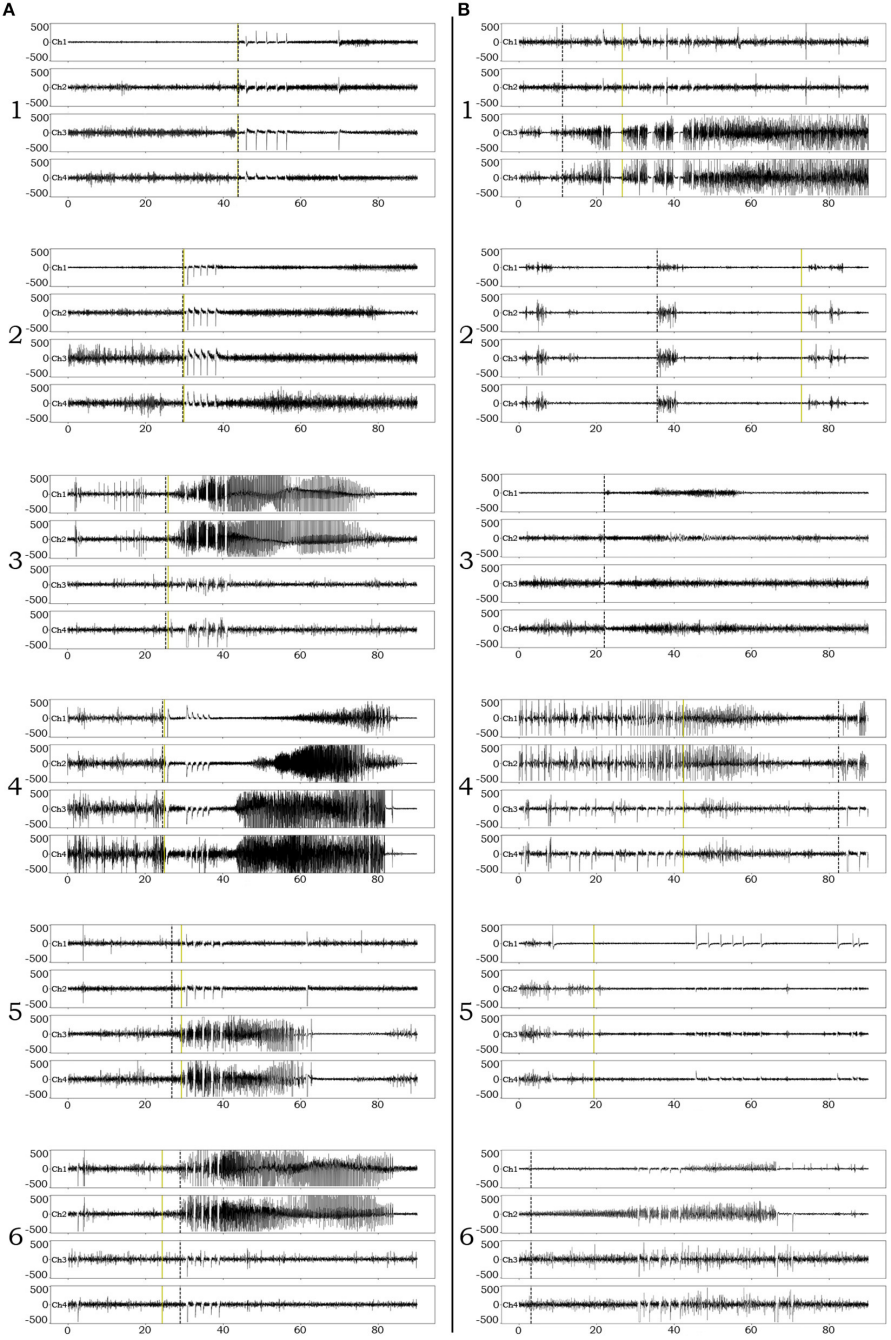
Examples of iEEG ictal pattern detection by the deep-learning neural network. Black dotted vertical lines represent the ictal pattern onset marking set by expert neurophysiologists. Green dotted vertical lines represent the CNN's annotation. **(A)** Examples of successful identifications of RNS ictal patterns with variable degrees of onset accuracy (1–6). **(B)** Examples of unsuccessful identifications. 1. Although the CNN classifies an ictal pattern in the epoch file, the actual onset is missed due to the presence of semi-regular brief diffuse electro-decrements at the beginning of the ictal pattern. 2. Although the CNN classifies an ictal pattern in the epoch file, the marker is placed at the onset of brief interictal activity that resembles the actual, also brief, ictal pattern. 3. Ictal pattern taking place in a single channel, in a patient with distant electrode implantation, is not acknowledged by the CNN (false negative). 4. Highly concentrated interictal activity is annotated by the CNN as ictal pattern (most of the times resulting in a false positive, unless an ictal pattern co-existed in the epoch file as in this example). 5. The recording occurred during the transition from sleep to wakefulness (arousal) and the CNN annotated the sudden introduction of normal background high frequencies as ictal pattern onset (false positive). 6. The CNN missed the ictal pattern for no apparent reason.

Examples of successful detections are presented in Figure 3A. In order to appreciate confounds that influenced accuracy and resulted in suboptimal detections, we performed manual review of failed detection items and identified 7 main categories of CNN pitfall conditions: (1) Ictal electro-decrements that reduce the signal amplitude to baseline levels (Figure 3B_1_). (2) Brief ictal patterns that can be confused for interictal bursts (Figure 3B_2_). (3) Ictal patterns isolated to a single channel (Figure 3B_3_). (4) Highly concentrated interictal activity (Figure 3B_4_). (5) Changes in the brain state in the context of the sleep-wake cycle (Figure 3B_5_). (6) Progressive modulation of electrographic ictal features [not shown, see (56)]. (7) Undetermined reasons (Figure 3B_6_).

## Discussion

This study describes a deep neural network that achieved high accuracy in seizure detection using a large dataset of expert-validated ictal patterns from the iEEG recordings of RNS-implanted epilepsy patients. The large size of our dataset allowed us to test two scenarios: (1) to evaluate seizure detection in an existing collection of recordings (including a random selection of the patient's ictal patterns in the training dataset) (scenario 1), and (2) to evaluate seizure detection on a prospective basis for new patients (including the earliest consecutive recorded ictal patterns of the patient in the training dataset) (scenario 2). We performed our evaluations using hold-one-patient-out cross validation. Specifically, the model was trained on 22 patients and evaluated in 12/22 patients for which at least > 35 ictal patterns were available, in a hold-one-patient-out cross validation fashion. For the “chronic recording scenario” (scenario 1), the model was trained on 22 patients, 0–30 seed examples from the test patient chosen uniformly at random, and it was evaluated on the remaining examples for the test “hold-one-out” patient. For the “recent recording scenario” (scenario 2), the model was also trained on 22 patients and the 0–30 seed examples were chosen to be the earliest possible recordings for the test patient, to evaluate the ability of the model to predict future examples for that patient. In turn, we report average results over all possible “hold-one-out” patients (12 total, after excluding patients with fewer than 35 ictal events). Our deep learning architecture achieved accuracy comparable to experts in both clinically relevant scenarios (0.84 and 0.80, respectively) using limited seed datasets containing 30 random and 20 consecutive ictal patterns, respectively. In the only previous report investigating the inter-rater reliability of RNS-derived intracranial seizure detection by experts (66), a manual review of 7,221 RNS-recorded electrographic epileptic events from 22 patients, experts reached an overall 0.79 agreement.

We also observed that AUPRC accuracy between just 5 vs. 10 seeds increased significantly, and although we obtained maxima at 20 and 30 seeds, the difference between 10, 20, and 30 seeds was not clinically meaningful. The standard procedure following RNS implantation requires 3–4 weeks of recording without therapeutic stimulation (baseline period) in order to collect ictal events and manually tune the on-board RNS detectors (51–54). Our model's training data requirements fit nicely with the RNS procedure, and the CNN could be used to improve the device's event detection capabilities in real-time. Specifically, it could use the baseline seizure data for training to improve the overall detection accuracy, and greatly reduce the need for the current practice of repeated heuristic manual adjustments of detection parameters (67).

Finally, we observed that although there was no statistically significant difference in ictal pattern identification between implanted anatomy groups, ictal patterns in developmental malformations and the hippocampus were more reliably classified than neocortical patterns. For that reason, we performed manual review of failed classifications and determined several systematic causes that turned out to have negatively affected the ictal pattern onset accuracy, although the mean values were well within the pre-determined tolerance window. Most failures and misses that we identified and hereby report have a neurophysiological rather than a computational background, comprised of a constellation of patterns that have often raised concerns within the epilepsy community (68, 69): patterns of interictal activity (70, 71), ictal electrodecrement patterns (72), iEEG patterns during the shift from sleep to wakefulness (73) and vice-versa (74), brief and regionally isolated ictal patterns (75), as well as the recently highlighted effect of ictal pattern modulation due to prolonged stimulation (56, 76). The overall lack of major confounds related to RNS anatomical substrates, suggests that the variety introduced by the anatomical origin of ictal patterns is unlikely to interfere with deep learning and performance.

We took several measures to quantify and reduce model overfitting. First, we report cross-validated results wherein the model is evaluated on different recordings than which it is trained on. Also, we did not extensively or automatically tune hyperparameters. For example, our learning rate varies from 0.1 to 0.025 and we train for exactly 20 epochs. Finally, we trained with both batch normalization (62) and dropout (77) that have experimentally been shown to act as regularizers.

The use of this CNN as an off-line tool can have an important impact in the routine clinical evaluation of epileptic patients implanted with RNS. Due to its high reliability in detecting ictal patterns, our tool can reflect an accurate overview of the patient's progress with neurostimulation therapy and support further quantitative assessments (57). Improvements in accuracy of seizure detection can also identify potential breakthrough seizures early enough for the physician to adjust and adapt the treatment strategy and achieve better seizure control, reducing thereby the risk of life-threatening emergencies such as status epilepticus and sudden unexpected death in epilepsy (54, 78).

We developed and presented a deep learning neural network that performs detection of RNS-derived ictal patterns with the highest published accuracy to date. The key to its performance is the large training dataset that allows the network to develop expertise; a pool of data that only the RNS device can provide due to its ability to sample and record neural activity over long periods of time. We are confident that this technology will improve the management of RNS patients and become pivotal for applications requiring high accuracy in intracranial seizure detection.

## Data Availability Statement

The raw data supporting the conclusions of this article will be made available by the authors, without undue reservation.

## Ethics Statement

The studies involving human participants were reviewed and approved by Mass General Brigham Human Research Protection Committee. Written informed consent for participation was not required for this study in accordance with the national legislation and the institutional requirements.

## Author Contributions

AC, NS, RMR, and VK designed the study and interpreted data. AC and NS organized the RNS data from the clinical system, created the figures, and result metrics. AC created the convolutional neural network and performed the experiments. NZ and VK reviewed and marked seizure patterns in the RNS-derived iEEG data. AU directed the RNS clinic and managed patient compliance with clinical data uploads. RMR performed the RNS implantations. All authors read and approved the submitted manuscript.

## Conflict of Interest

RMR has served as a consultant for NeuroPace, Inc. The remaining authors declare that the research was conducted in the absence of any commercial or financial relationships that could be construed as a potential conflict of interest.

## Supplementary Material

Supplementary Figure 1 RNS iEEG data acquisition. a. Pre-operative MRI and post-implantation CT fused image aligned in the axial plane across the trajectory of the implanted RNS lead in the left hippocampus of patient 6. b. iEEG data from the baseline period. The two distal anterior hippocampal contacts make bipolar channel 1, and the two proximal posterior hippocampal ones make bipolar channel 2, that record a unilateral iEEG seizure pattern in the left hippocampus during the baseline period starting in channel 2 (onset at red line). c. Respective iEEG data from the 1st programming epoch where stimulation was activated. During stimulation the amplifier is disconnected, thereby generating a rectangular pulse artifact in the time domain. d, e, and f show the respective imaging and data for an independent right hippocampal electrographic seizure pattern starting in channel 3 of the same patient.
