# Supplementary figures and images for: Expert-Level Intracranial Electroencephalogram Ictal Pattern Detection by a Deep Learning Neural Network
Source: Front Neurol. 2021 May 3;12:603868. doi: 10.3389/fneur.2021.603868 (PMC8126697; doi:10.3389/fneur.2021.603868)

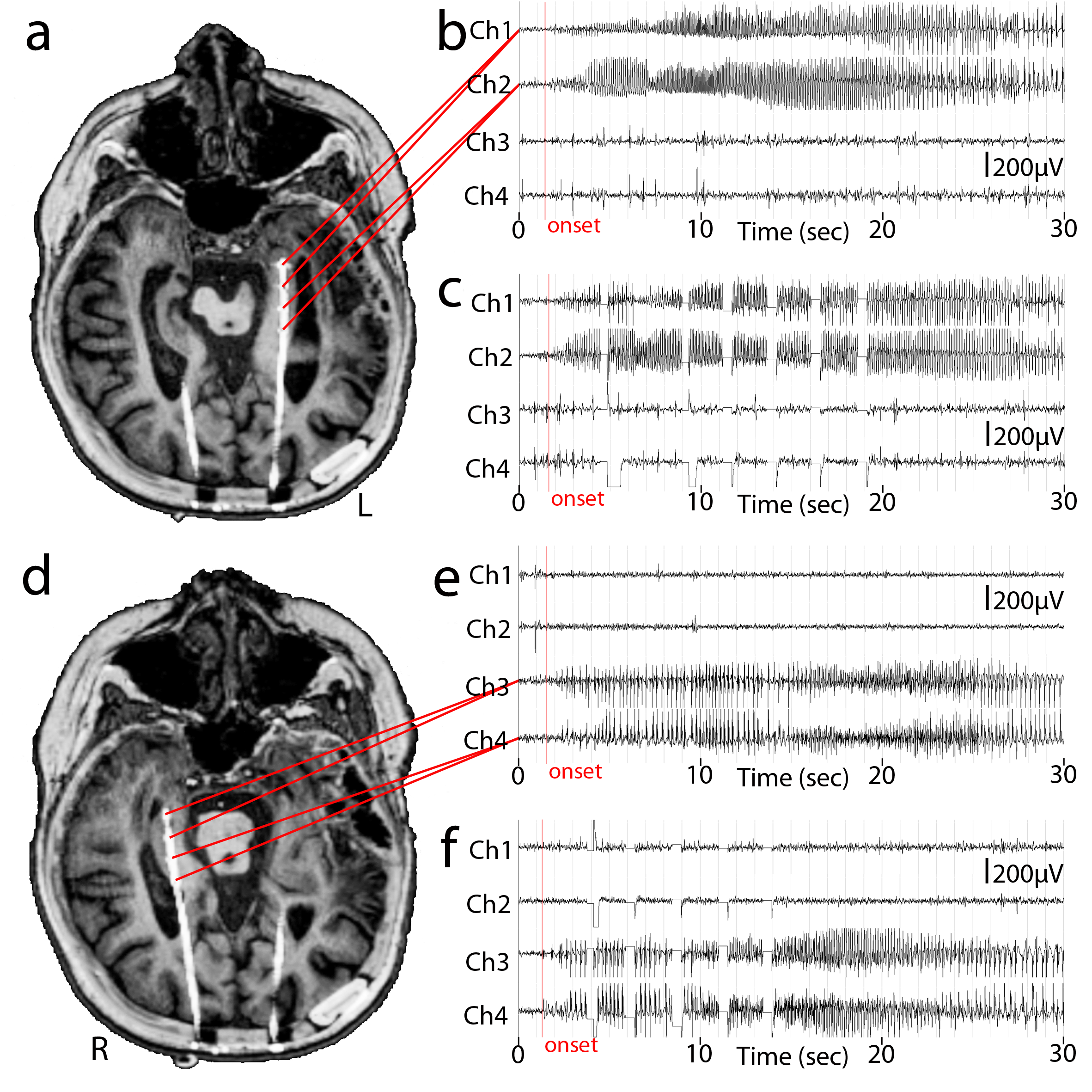

Supplement: Supplementary Figure 1 — RNS iEEG data acquisition. a. Pre-operative MRI and post-implantation CT fused image aligned in the axial plane across the trajectory of the implanted RNS lead in the left hippocampus of patient 6. b. iEEG data from the baseline period. The two distal anterior hippocampal contacts make bipolar channel 1, and the two proximal posterior hippocampal ones make bipolar channel 2, that record a unilateral iEEG seizure pattern in the left hippocampus during the baseline period starting in channel 2 (onset at red line). c. Respective iEEG data from the 1st programming epoch where stimulation was activated. During stimulation the amplifier is disconnected, thereby generating a rectangular pulse artifact in the time domain. d, e, and f show the respective imaging and data for an independent right hippocampal electrographic seizure pattern starting in channel 3 of the same patient. [file Image_1.TIF]
